# Usefulness of Serum Calcium in the Risk Stratification of Midterm Mortality among Patients with Acute Coronary Syndrome

**DOI:** 10.1155/2019/9542054

**Published:** 2019-11-03

**Authors:** Xingbo Gu, Xiaotong Ding, Hongna Sun, Ningning Chen, Dandan Liu, Dianjun Sun, Shu Wang

**Affiliations:** ^1^Department of Cardiology, The First Affiliated Hospital, Cardiovascular Institute, Harbin Medical University, Harbin 150001, China; ^2^Center for Endemic Disease Control, Chinese Center for Disease Control and Prevention, Harbin Medical University, Harbin 150081, China

## Abstract

Serum calcium has been reported to be a predictor of short-term prognosis; however, evidence regarding its association with midterm mortality is scarce. We investigated the association between serum calcium levels at admission and midterm mortality in a retrospective cohort of 2594 consecutive patients with acute coronary syndrome (ACS) who presented to the First Affiliated Hospital of Harbin Medical University from November 2014 to December 2016. Patients were assigned to 4 groups according to the quartiles of serum calcium levels (Ca-*Q*1–4) and were followed longitudinally for the time to all-cause death. During a median follow-up period of 21.8 months (17.5∼29.5, IQR), 124 patients died (4.8%) of all causes. Kaplan–Meier curves showed that the incidence of midterm mortality differed significantly (log-rank *P*=0.038) among the quartiles of serum calcium levels at admission. After adjustment for the confounders that were significant in the univariate analysis, the hazard ratios for the lowest quartile of serum calcium was 1.86 (95% CI, 1.05–3.31; *P*=0.033), compared with the third quartile (reference group). A multiple restricted cubic spline regression model suggested a reverse J-shaped association between serum calcium levels and midterm mortality, and the lowest risk of mortality was associated with approximately 2.32 mmol/l of serum calcium. In conclusion, the serum calcium level is an independent predictor of all-cause midterm mortality among ACS patients. Patients with abnormal serum calcium levels at admission need more targeted treatments.

## 1. Introduction

Acute coronary syndrome (ACS) continues to be the leading cause of morbidity and mortality worldwide, costing billions of dollars in medical costs and depleting many public health resources [1]. However, the mechanisms underlying the poor prognosis of ACS patients have not yet been well elucidated, and it may be affected by several factors, such as baseline characteristics and treatment before, during, and after hospitalization [2]. Thus, it is still not possible to accurately identify the patients who are most likely to experience poor outcomes.

Electrolytes play a critical role in maintaining homeostasis, regulating heart and neurological functions, fluid balance, oxygen delivery, and acid-base balance. Serum calcium ions are considered to be one of the major electrolytes associated with the electrophysiological properties of the myocardium [3]. Calcium is involved in the physiological and biochemical processes of heart electrophysiology and contraction, blood coagulation, neurotransmitter release, enzyme activity regulation, and blood pressure regulation [4, 5]. Several previous studies have shown that a decreased or/and increased serum calcium level is an independent risk factor for in-hospital mortality in patients with acute myocardial infarction [6–10]. However, the clinical outcomes of these studies were mainly in-hospital death or 30-day postdischarge death, and evidence regarding the association between serum calcium levels and midterm mortality in ACS patients is still scarce. Therefore, whether the serum calcium level is an independent risk factor for midterm mortality or just an intermediate variable that is associated with other cardiovascular risk factors and clinical outcomes remains unknown.

In the present study, we investigated the possible association between serum calcium levels and midterm mortality in a large cohort of ACS patients in China.

## 2. Materials and Methods

### 2.1. Study Design and Population

**Figure 1:**
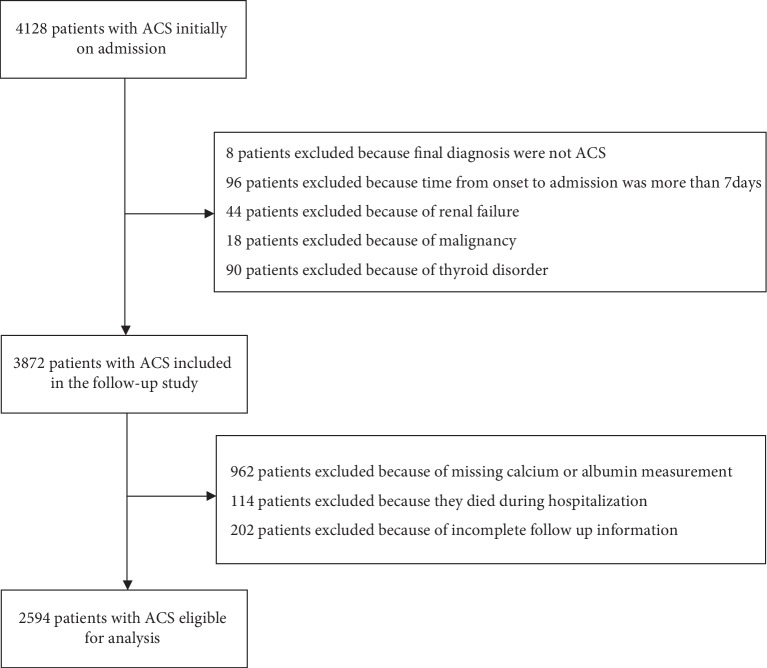
Patient flow chart.

The present investigation was a single-center, observational, retrospective cohort study in China. From November 2014 to December 2016, we identified 4,128 unique, consecutive patients with ACS who were admitted to the Department of Cardiology of the First Affiliated Hospital of Harbin Medical University. Patients aged ≥18 years with a clinical diagnosis of ACS who underwent an assessment of serum calcium levels were included in this study. ACS was diagnosed according to the criteria defined by the World Health Organization, based on patient medical history, clinical symptoms, electrocardiogram, echocardiography, and the levels of cardiac enzymes. Additional exclusion criteria were as follows: (1) final diagnosis were not ACS; (2) time from onset to admission more than 7 days; (3) with known renal failure, malignancy, and thyroid disorder. A total of 3,872 patients were potentially eligible for this study. In addition, we further excluded 1,278 patients because of missing calcium or albumin measurement (*n* = 962), died during hospitalization (*n* = 114), incomplete follow-up information (*n* = 202). Finally, a total of 2,594 patients with a diagnosis of ACS were eligible for this analysis (Figure 1 shows the flow chart of patients). The ethics committee of the First Affiliated Hospital of Harbin Medical University approved the study protocol.

### 2.2. Baseline Data Collection and Serum Calcium

A team of trained cardiologists, postgraduates, and statistical analysts reviewed the eligibility of study participants and collected the data. Demographic data, medical history, medication use, in-hospital complications, reperfusion strategy, and the results of laboratory and echocardiography tests were obtained from the electronic medical records. Venous blood samples were obtained after overnight fasting from each patient within the first 24 h of admission. Laboratory variables were examined at the hospital's clinical laboratories. Plasma ion concentrations, including serum calcium levels, were measured using an ion-specific electrode (Auto Analyser; Hitachi Inc., Tokyo, Japan). The estimated glomerular filtration rate (eGFR) was calculated using the serum creatinine level with the CKD-EPI China equation with an adjusted coefficient of 1.1 for the Chinese population [11]. The first available serum calcium (mmol/L) levels were corrected for albumin levels (g/dL) according to the following formula: observed serum calcium (mmol/L) + 0.02 *∗* (40 g/L− albumin g/L) [12]. All serum calcium levels for each patient were corrected for albumin and categorized into 4 groups according to the quartiles of serum calcium concentrations: *Q*1 (<2.21 mmol/l), *Q*2 (2.21∼2.28mmol/l), *Q*3 (2.28∼2.36 mmol/l), and *Q*4 (>2.36 mmol/l) for all analyses.

### 2.3. Primary Endpoint and Clinical Follow-up

The primary endpoint for this study was midterm all-cause mortality (within 3 years after discharge or until February 2018). Clinical follow-up endpoint data and the timing of clinical outcomes were obtained by reviewing outpatient electronic medical records and telephone interviews. Mortality data and the details of deaths were available from the medical records of patients or telephone contact with their family members.

### 2.4. Statistical Analysis

The data distribution was examined using the Kolmogorov–Smirnov test. Continuous but nonnormally distributed variables are presented as medians (interquartile range), and categorical variables are presented as counts and percentages. Baseline characteristics (continuous variables) among the groups of quartiles of serum calcium were compared using the nonparametric Kruskal–Wallis test, and categorical variables were tested using the chi-square test or Fisher's exact test, as appropriate. The freedom from midterm mortality according to the quartiles of serum calcium was estimated using the Kaplan–Meier method and compared using the log-rank test. Univariate and multivariate Cox proportional hazards regression models were used to estimate the risk of midterm mortality. Hazard ratios (HRs) and confidence intervals (CIs) were calculated for the quartiles of serum calcium concentrations, with the third quartile (*Q*3) as the reference. The association between serum calcium levels and midterm mortality was evaluated in 3 models. The crude and age- and sex-adjusted association models were established as Model 1 and Model 2, respectively. For the multivariate adjusted Model (Model 3), only the potential covariate variables that were significantly associated with midterm mortality in the univariate analyses were included. In addition, we performed restricted cubic splines in multivariate adjusted Cox models to evaluate the shape of the association between serum calcium levels (continuous) and midterm mortality. The C-index and net reclassification improvement (NRI) were calculated to examine the independent predictive value of serum calcium levels for midterm mortality. The C-index was defined as the area under the receiver operating characteristic curves between individual predictive probabilities for events. NRI was determined to calculate the percentage of individuals who would be correctly reclassified when the existing model was updated by the inclusion of the serum calcium concentration. To examine the potential heterogeneous effect of serum calcium on midterm mortality, we performed subgroup analyses. Subgroup analyses were conducted in a multivariate adjusted model stratified by age (≥60 vs. 60 years), sex, current smoking, history of hypertension, history of stroke, history of coronary heart disease, classification of ACS, eGFR (≥60 vs. <60 ml/min/1.73 m^2^), and LVEF (≥50 vs. <50%). The first-order interactions in multivariable Cox proportional hazards regression models were examined by entering interaction terms between the serum calcium and the subgroup variables. And we assess the effect of serum calcium on midterm mortality in each subgroup. The R (version 3.4.1) software was used for managing the data, generating plots, and performing the statistical analyses. Two-sided *P* values < 0.05 were considered statistically significant for all estimates.

## 3. Results

### 3.1. Clinical Characteristics

Among the 2594 patients with ACS who met the diagnostic criteria for this study, the median age was 60.0 years (53.0∼68.0, IQR), and 68.4% of the patients were men. Serum calcium concentrations approximated a normal distribution ([Supplementary-material supplementary-material-1]), with a mean level of 2.27 (0.46, SD) and a median level of 2.28 (2.21∼2.36, IQR). The baseline characteristics of the patients based on serum calcium quartiles are listed in Table 1. In the low serum calcium group, the majority of patients were men who had ST-elevation myocardial infarction (STEMI), with lower average hemoglobin, uric acid, serum phosphate, and serum chloride levels; lower LVEF; and higher albumin, BUN, serum magnesium, and LAD levels.

### 3.2. Clinical Outcomes

**Figure 2:**
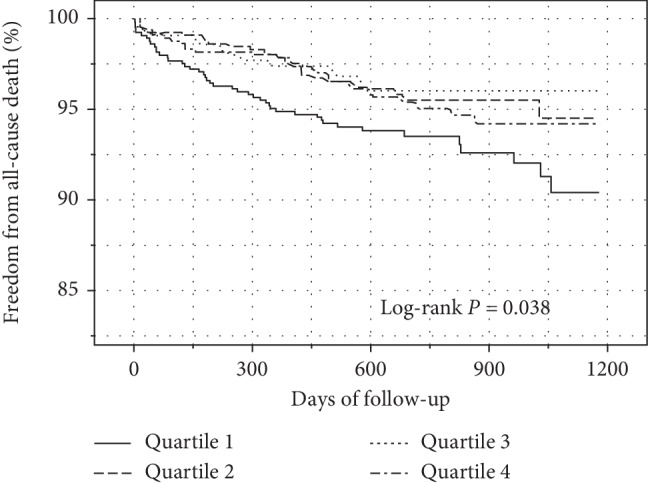
Kaplan–Meier curves comparing survival among serum calcium quantiles in patients with acute coronary syndrome (ACS). The curves of the quartiles of calcium differed significantly (log-rank *P*=0.038), and patients in the lowest calcium quartile had the highest cumulative incidence of mortality.

Over the median follow-up period of 21.8 months (17.5∼29.5, IQR), 124 patients died (4.8%) due to all causes. Patients in the lowest serum calcium quartile exhibited the highest incidence of mortality ([Supplementary-material supplementary-material-1]). Kaplan–Meier curves for the freedom from all-cause death according to the quartiles of serum calcium are shown in Figure 2. The curves of the quartiles of calcium differed significantly (log-rank *P*=0.038), and patients in the lowest calcium quartile had the highest cumulative incidence of mortality. Univariate Cox proportional hazards regression indicated that age, sex, history of hypertension, history of stroke, history of coronary heart disease, complicated heart failure, complicated arrhythmia, complicated atrioventricular block, aspirin on admission, percutaneous coronary intervention (PCI), coronary angiography, hemoglobin level, uric acid level, estimated glomerular filtration rate (eGFR), serum phosphate level, serum magnesium level, serum potassium level, left ventricular ejection fraction (LVEF), left atrial diameter (LAD), left ventricular end diastolic diameter (LVEDD), interventricular septum thickness (IVST), and serum calcium level were significantly associated with midterm mortality (all *P* < 0.05, [Supplementary-material supplementary-material-1]).

**Figure 3:**
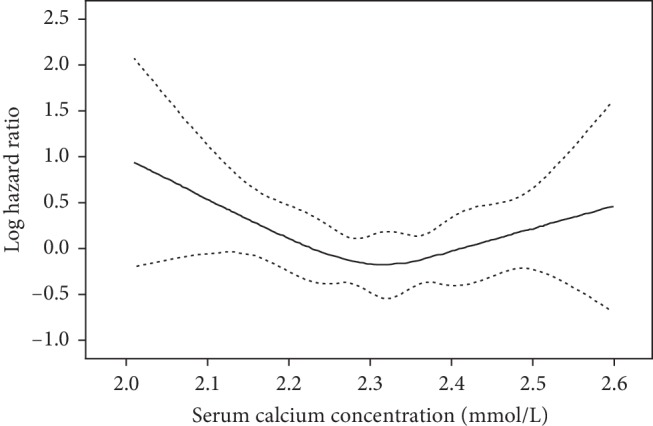
Multiple spline regression analyses of log hazard ratios (solid line) and their 95% CIs (dotted line) of midterm mortality associated with serum calcium levels in patients with acute coronary syndrome (ACS). The relationship between serum calcium and midterm mortality was reverse J-shaped.

Table 2 shows the different Cox proportional hazard models for serum calcium levels and midterm mortality. In the unadjusted (Model 1) and age- and sex-adjusted (Model 2) models, the hazard ratio for midterm mortality was significantly lower among patients in the lowest quartile (*Q*1, <2.21 mmol/l) of serum calcium levels compared than among those in the third quartile (*Q*3, 2.28∼2.36 mmol/l). After adjustment for other confounders (significant factors in the univariate analysis), the hazard ratios for midterm mortality among patients in the lowest quartile of serum calcium levels was 1.86 (95% CI, 1.05–3.31; *P*=0.033), compared with the third quartile (Model 3). In addition, we found that in any of these models, there were statistically significant differences among patients in the lowest quartile and second quartile (*Q*2, 2.21∼2.28 mmol/l), but no statistically significant differences when compared patients in the lowest quartile and highest quartile of serum calcium levels (*Q*4, >2.36 mmol/l). When using a multiple restricted cubic spline Cox regression model, the result showed that the relationship between serum calcium and midterm mortality was seemed reverse J-shaped, and we also observed that the lowest risk of mortality was associated with approximately 2.32 mmol/l serum calcium (Figure 3).

**Figure 4:**
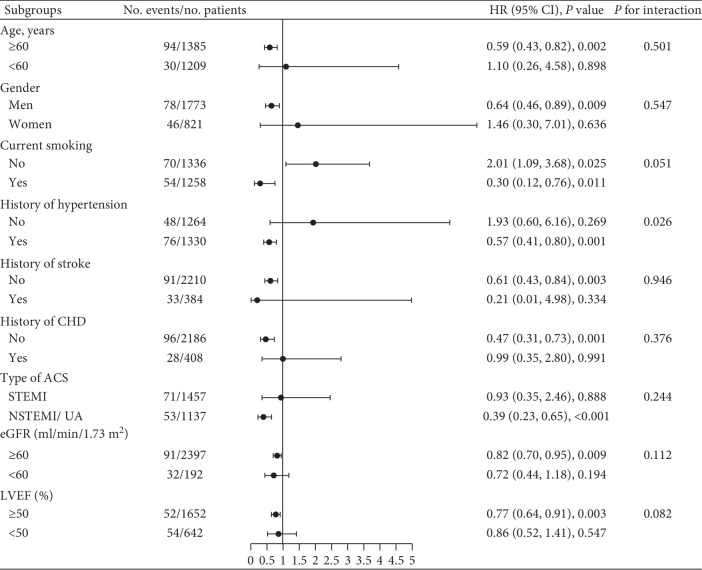
Forest plots of the subgroup analysis. There was a significant interaction between a history of hypertension and serum calcium levels with regard to the risk of midterm mortality (*P* -interaction = 0.026).

[Supplementary-material supplementary-material-1] shows that the C-index for midterm mortality was higher in the established risk factors model after the addition of serum calcium (0.75 vs. 0.77; *P*=0.042), and the NRI for midterm mortality also increased after serum calcium was added to the established risk factors model (net reclassification indexes, 0.22; *P*=0.025). The subgroup analysis showed that there was no effect modification of the association between serum calcium levels and midterm mortality in different groups of age, sex, current smoking, history of stroke, history of coronary heart disease, classification of ACS, eGFR, and LVEF (Figure 4). However, there was a significant interaction between the history of hypertension and serum calcium levels with regard to the risk of midterm mortality (*P* -interaction = 0.026). The significant association between serum calcium and mortality was only observed among patients with a history of hypertension. Kaplan–Meier curves for comparing survival among serum calcium quantiles in patients with or without a history of hypertension are shown in [Supplementary-material supplementary-material-1].

## 4. Discussion

The major findings of the present study of 2594 patients were that the serum calcium level is an independent predictor of the all-cause midterm mortality risk in ACS patients, and a reverse J-shaped relationship between serum calcium levels and mortality was observed. Patients with reduced serum calcium levels had a significantly higher incidence of midterm mortality, and the lowest risk of mortality was associated with approximately 2.32 mmol/l serum calcium. This relationship remained consistent in different adjusted models. Serum calcium levels added to the established risk factors model could improve the prediction of midterm mortality. Furthermore, the subgroup analyses indicated that serum calcium and hypertension history had a significant interaction with regard to mortality prediction, and serum calcium levels were predictors of mortality only in patients with a history of hypertension.

To the best of our knowledge, this is the first study to show that serum calcium levels are associated with the midterm mortality risk in ACS patients. It is well known that increased serum calcium levels are associated with the risk of cardiovascular events and mortality in the general population [13–18]. Nevertheless, evidence is inconsistent in most hospital-based studies regarding the relationship between serum calcium levels and mortality in patients with cardiac and noncardiac diseases [19–21]. Two studies in China reported that decreased baseline serum calcium levels were associated with a high risk of in-hospital mortality in myocardial infarction patients [6, 9]. A recent study with a large sample size reported a *U*-shaped association between serum calcium levels and in-hospital mortality [8]. However, these studies were mainly focused on the short-term outcome of patients, and evidence regarding the midterm outcome of patients is very scarce. An earlier prospective study of stable coronary heart disease patients suggested that a higher baseline calcium level was associated with the risks of cardiovascular and all-cause mortality [22]. One study in southern China reported for the first time that lower serum calcium levels at the baseline were correlated with an increased risk of long-term all-cause and cardiovascular mortality among coronary heart disease patients, while higher serum calcium levels (≥2.37 mmol/L) were not [7]. The potential explanation for the discrepancies among these studies may be due to differences in populations, the clinical characteristics of the patients, the inclusion and exclusion criteria, the study sample size, and the length of follow-up. In the present study of ACS patients, after adjusting for demographic characteristics, medical history, medication and treatment at admission, and other conventional cardiovascular risk factors, low serum calcium levels at admission were found to increase the midterm mortality risk. Moreover, although there was no statistical difference between the high serum calcium group and the reference group, it is still seen from the relationship curve that patients with high serum calcium had an upward trend of death. Interestingly, there is still no consensus on the cutoff points to define the levels of serum calcium needed to reduce the risk of death in most studies. Our findings suggested that maintaining serum calcium levels at approximately 2.32 mmol/l at the time of admission can minimize the midterm risk of death. Furthermore, some previous clinical studies indicated that altered calcium homeostasis was associated with an increased mortality risk in patients with chronic kidney disease and heart failure [19, 20, 23]. In this study, we excluded patients with renal failure and adjusted for the eGFR and cardiac function indicators in the multivariate analysis. In addition, we stratified patients on the basis of the eGFR, LVEF, and other important clinical features and ultimately demonstrated the effectiveness of serum calcium levels at admission in predicting midterm mortality risk among ACS patients.

The exact mechanisms through which serum calcium leads to an elevated risk of midterm death remain unclear. Calcium ions are one of the most critical signal transduction molecules in cells and play an indispensable role in cardiac electrophysiological processes [24]. The destruction of intracellular calcium homeostasis seriously affects the cardiac excitation-contraction coupling function, and severe cases can lead to cardiac death [25]. Decreased serum calcium levels lead to an abnormal increase in intracellular calcium levels, which may accelerate the formation of atherosclerotic plaques and increase cardiotoxicity [26, 27]. In addition, serum calcium levels may cause L-type calcium channel inactivation, leading to platform depolarization and the shortening of the cardiac action potential, causing a prolonged QT interval, which is known to be associated with cardiac death in the general population [28]. High serum calcium levels can also cause an increase in free calcium levels, disrupting calcium homeostasis and interacting with phosphate, vitamin D, and other metabolites to accelerate the progression of calcification, ultimately leading to the occurrence of acute cardiovascular events [29, 30]. Furthermore, decreased serum calcium is associated with various well-known cardiovascular risk profiles, such as hypertension, left ventricular hypertrophy, arrhythmia, and heart failure [24]. The present study shows that the relationship between midterm mortality and serum calcium levels is more obvious in patients with a history of hypertension.

There are several limitations in our study. First, we only evaluated serum calcium at the time of admission, and serum calcium levels may vary over time. This prevented us from assessing the relationship between changes in serum calcium levels over time and the risk of midterm clinical outcomes. Second, some seriously ill patients with severe imbalances in calcium homeostasis died on their way to the hospital. We did not have information on these patients, which may have led to the underestimation of the association. Third, the study outcome only focused on all-cause midterm death; therefore, our results do not enable us to explain the associations with cause-specific death and other clinical outcomes. Fourth, the characteristics of the excluded patients who were lost to follow-up or lacked the measurement of serum calcium may be different from those of the selected patients, which could have resulted in research bias. Fifth, although we collected numerous relevant covariates, some potential confounders (e.g., vitamin D, bone metabolites, and thyroid hormones) were not considered. Finally, this study was conducted at a single center with a relatively high level of cardiovascular disease treatment and only involved Chinese patients; thus, the results of this work may not be applicable to all populations.

## 5. Conclusions

Serum calcium levels at the time of admission are an independent predictor of all-cause midterm mortality among ACS patients, with a reverse J-shaped relationship. The effective use of serum calcium as an available biomarker may play a role in the midterm risk stratification of patients with ACS. Further studies are needed to determine how therapy affecting serum calcium levels can improve the midterm prognosis and to clarify the underlying mechanisms.

## Figures and Tables

**Table 1:** Characteristics of patients with acute myocardial infarction according to serum calcium quartiles.

Characteristics	Serum calcium concentration (mmol/l)				*P* value
	*Q*1 <2.21	*Q*2 2.21∼2.28	*Q*3 2.28∼2.36	*Q*4 >2.36	
Number of patients	645	648	648	653	

*Demographics*					
Age (years)	61.0 (53.0∼69.0)	61.0 (53.0∼68.0)	60.0 (53.0∼67.0)	59.0 (52.0∼66.0)	0.226
Men (*n*, %)	208 (32.2)	193 (29.8)	187 (28.9)	233 (35.7)	0.039
Current smoking (*n*, %)	306 (47.4)	321 (49.5)	312 (48.1)	319 (48.9)	0.889
Current alcohol use (*n*, %)	126 (19.5)	119 (18.4)	128 (19.8)	139 (21.3)	0.620

*Medical history*					
Hypertension (*n*, %)	316 (49.0)	346 (53.4)	332 (51.2)	336 (51.5)	0.472
Diabetes mellitus (*n*, %)	135 (20.9)	131 (20.2)	141 (21.8)	163 (25.0)	0.172
Stroke (*n*, %)	88 (13.6)	115 (17.7)	91 (14.0)	90 (13.8)	0.113
CHD (*n*, %)	93 (14.4)	110 (17.0)	100 (15.4)	105 (16.1)	0.637
Previous PCI (*n*, %)	34 (5.3)	48 (7.4)	59 (9.1)	46 (7.0)	0.067

*In-hospital complications*					
Acute heart failure (*n*, %)	125 (19.4)	111 (17.1)	115 (17.7)	90 (13.8)	0.054
Acute arrhythmia (*n*, %)	20 (3.1)	18 (2.8)	22 (3.4)	5 (0.8)	0.010
AV block (*n*, %)	41 (6.4)	43 (6.6)	34 (5.2)	34 (5.2)	0.585

*Medication on admission*					
ACEI/ARB (*n*, %)	213 (33.0)	234 (36.1)	241 (37.2)	245 (37.5)	0.316
Beta-blocker (*n*, %)	364 (56.4)	381 (58.8)	376 (58.0)	390 (59.7)	0.670
Aspirin (*n*, %)	602 (93.3)	611 (94.3)	622 (96.0)	627 (96.0)	0.070
Statin (*n*, %)	563 (92.0)	576 (93.1)	588 (93.3)	604 (94.5)	0.359

*Main diagnosis*					
STEMI (*n*, %)	385 (59.7)	386 (59.6)	359 (55.4)	327 (50.1)	0.001
NSTEMI/UA (*n*, %)	260 (40.3)	262 (40.4)	289 (44.6)	326 (49.9)	

*Reperfusion strategy*						
Coronary angiography (*n*, %)	495 (76.7)	493 (76.1)	479 (73.9)	497 (76.1)	0.657
PCI (*n*, %)	476 (76.5)	464 (74.2)	441 (70.9)	469 (73.6)	0.160
Thrombolysis (*n*, %)	35 (5.4)	34 (5.2)	35 (5.4)	21 (3.2)	0.178

*Laboratory results*					
Hemoglobin (g/l)	140.2 (125.9∼152.2)	142.5 (130.6.∼154.5)	144.0 (133.0∼155.2)	144.7 (130.8∼156.8)	<0.001
Albumin (g/l)	41.2 (41.2∼43.9)	41.2 (40.2∼42.1)	41.2 (39.8∼41.8)	41.2 (39.2∼41.2)	<0.001
BUN (mmol/l)	5.5 (4.4∼6.8)	5.4 (4.4∼6.6)	5.5 (4.4∼6.6)	5.4 (4.4∼6.8)	0.027
Fasting glucose (mmol/l)	6.1 (5.2∼7.6)	5.9 (5.1∼7.7)	6.1 (5.2∼7.8)	6.0 (5.1∼8.0)	0.557
eGFR (ml/min/1.73 m^2^)	104.1 (87.9∼114.0)	102.0 (86.5∼112.6)	102.3 (88.2∼111.9)	102.2 (85.1∼112.3)	0.877
Uric acid (*μ*mol/l)	310.8 (255.2∼379.6)	329.7 (265.2∼387.3)	330.2 (274.8∼402.0)	332.4 (277.0∼396.4)	0.002
Serum phosphate (mmol/l)	1.1 (1.0∼1.3)	1.1 (1.0∼1.3)	1.2 (1.0∼1.3)	1.2 (1.1∼1.4)	0.011
Serum magnesium (mmol/l)	0.9 (0.8∼0.9)	0.9 (0.8∼0.9)	0.9 (0.8∼0.9)	0.9 (0.8∼0.9)	<0.001
Serum potassium (mmol/l)	4.1 (3.8∼4.3)	4.1 (3.9∼4.4)	4.2 (3.9∼4.5)	4.2 (3.9∼4.5)	0.220
Serum sodium (mmol/l)	139.8 (136.8∼142.5)	140.4 (137.8∼143.1)	141.1 (138.7∼143.6)	140.9 (138.4∼143.7)	<0.001
Serum chloride (mmol/l)	102.9 (100.3∼105.4)	103.3 (101.0∼105.5)	103.0 (101.2∼105.2)	102.7 (100.5∼105.0)	0.037

*Echocardiography results*					
LVEF (%)	55.0 (47.0∼60.0)	54.0 (48.0∼60.0)	55.0 (48.0∼60.0)	56.0 (50.0∼64.0)	<0.001
LAD (mm)	37.0 (34.0∼39.0)	36.0 (34.0∼39.0)	37.0 (35.0∼39.0)	36.0 (34.0∼38.0)	<0.001
LVEDD (mm)	50.0 (47.0∼53.0)	49.0 (47.0∼53.0)	50.0 (47.0∼53.0)	49.0 (46.0∼52.0)	0.014
IVST (mm)	9.6 (9.0∼10.2)	9.6 (9.0∼10.3)	9.6 (9.0∼10.4)	9.8 (9.0∼10.7)	0.146
LVPW (mm)	9.6 (9.0∼10.2)	9.6 (9.0∼10.2)	9.6 (9.0∼10.2)	9.7 (9.0∼10.4)	0.340

ACEI, angiotensin-converting enzyme inhibitors; ARB, angiotensin receptor blockers; BUN, blood urea nitrogen; CHD, coronary heart disease; eGFR, estimated glomerular filtration rate; IVST, interventricular septum thickness; LAD, left atrial diameter; LVEDD, left ventricular end diastolic diameter; LVEF, left ventricular ejection fraction; LVPW, left ventricular posterior wall thickness; NSTEMI, non-ST-segment elevation myocardial infarction; PCI, percutaneous coronary intervention; and STEMI, ST-segment elevation myocardial infarction. ^*∗*^Data are expressed as the mean (standard deviation) for normally distributed data, the median (interquartile range) for nonnormally distributed data, and the percentage (%) for categorical variables.

**Table 2:** Hazard ratios and 95% CIs of midterm mortality according to quartiles of serum calcium among patients with acute myocardial infarction.

	Serum calcium			
Model	*Q*1	*Q*2	*Q*3	*Q*4
	HR (95% CI)	HR (95% CI)	HR (95% CI)	HR (95% CI)
	*P*	*P*	*P*	*P*
Deaths/*N*	45/645	27/648	23/648	29/653
Model 1	1.89 (1.14, 3.12)	1.14 (0.65, 1.99)	Ref	1.22 (0.70, 2.10)
	0.013	0.646		0.482
Model 2	1.78 (1.08, 2.94)	1.14 (0.65, 1.98)	Ref	1.25 (0.72, 2.15)
	0.025	0.653		0.433
Model 3	1.86 (1.05, 3.31)	0.93 (0.49, 1.77)	Ref	1.48 (0.81, 2.70)
	0.033	0.833		0.206

Model 1: crude. Model 2: adjusted for age and sex. Model 3: adjusted for age (continuous), sex, history of hypertension, history of stroke, history of coronary heart disease, complicated heart failure, complicated arrhythmia, complicated atrioventricular block, aspirin on admission, percutaneous coronary intervention, coronary angiography, hemoglobin (continuous), uric acid (continuous), eGFR (continuous), serum phosphate (continuous), serum magnesium (continuous), serum potassium (continuous), LVEF (continuous), LAD (continuous), LVEDD (continuous), and IVST (continuous).

## Data Availability

The datasets used throughout this study are available from the corresponding author on reasonable request.

## Supplementary Materials

Supplementary Materials Supplemental Figure S1: distribution of serum calcium concentrations. Supplemental Figure S2: all-cause mortality rate based on the quantiles of serum calcium in patients with acute coronary syndrome (ACS). Patients in the lowest serum calcium quartile exhibited the highest incidence of mortality. Supplemental Figure S3: Kaplan–Meier curves comparing survival among serum calcium quantiles in acute coronary syndrome (ACS) patients with or without a history of hypertension. Supplemental Table S1: relationships between risk factors and midterm mortality in patients with acute coronary syndrome by univariate analysis. Supplemental Table S2: discriminatory ability of serum calcium for midterm mortality according to the C-Index and NRI.
