# Supplementary material for: Usefulness of Serum Calcium in the Risk Stratification of Midterm Mortality among Patients with Acute Coronary Syndrome
Source: Biomed Res Int. 2019 Nov 3;2019:9542054. doi: 10.1155/2019/9542054 (PMC6875235; doi:10.1155/2019/9542054)
Supplement: Supplementary Materials — Supplemental Figure S1: distribution of serum calcium concentrations. Supplemental Figure S2: all-cause mortality rate based on the quantiles of serum calcium in patients with acute coronary syndrome (ACS). Patients in the lowest serum calcium quartile exhibited the highest incidence of mortality. Supplemental Figure S3: Kaplan–Meier curves comparing survival among serum calcium quantiles in acute coronary syndrome (ACS) patients with or without a history of hypertension. Supplemental Table S1: relationships between risk factors and midterm mortality in patients with acute coronary syndrome by univariate analysis. Supplemental Table S2: discriminatory ability of serum calcium for midterm mortality according to the C-Index and NRI. [file 9542054.f1.docx]

**Supplementary Materials**


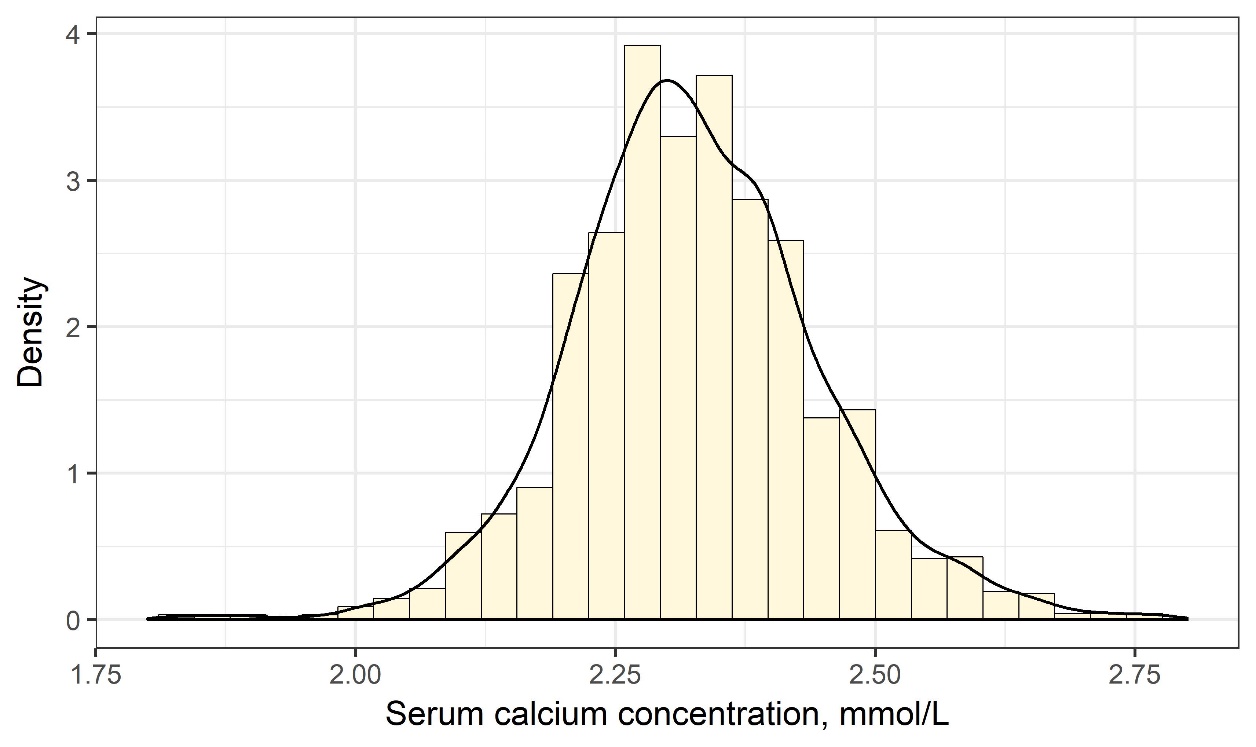


Supplemental Figure S1: Distribution of serum calcium concentrations. Serum calcium concentrations approximated a normal distribution with a mean level of 2.27 mmol/L (0.46, SD) and a median level of 2.28 mmol/L (2.21~2.36, IQR).


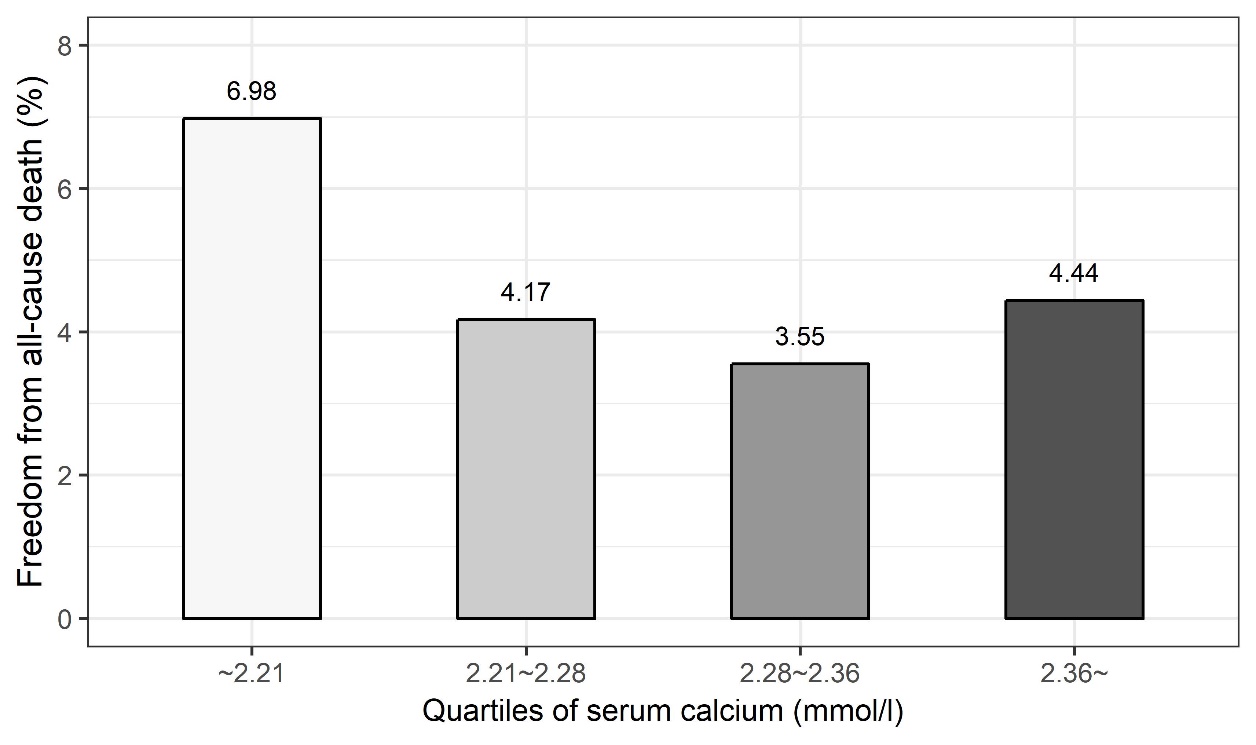


Supplemental Figure S2: All-cause mortality rate based on the quantiles of serum calcium in patients with acute coronary syndrome (ACS). Patients in the lowest serum calcium quartile exhibited the highest incidence of mortality.


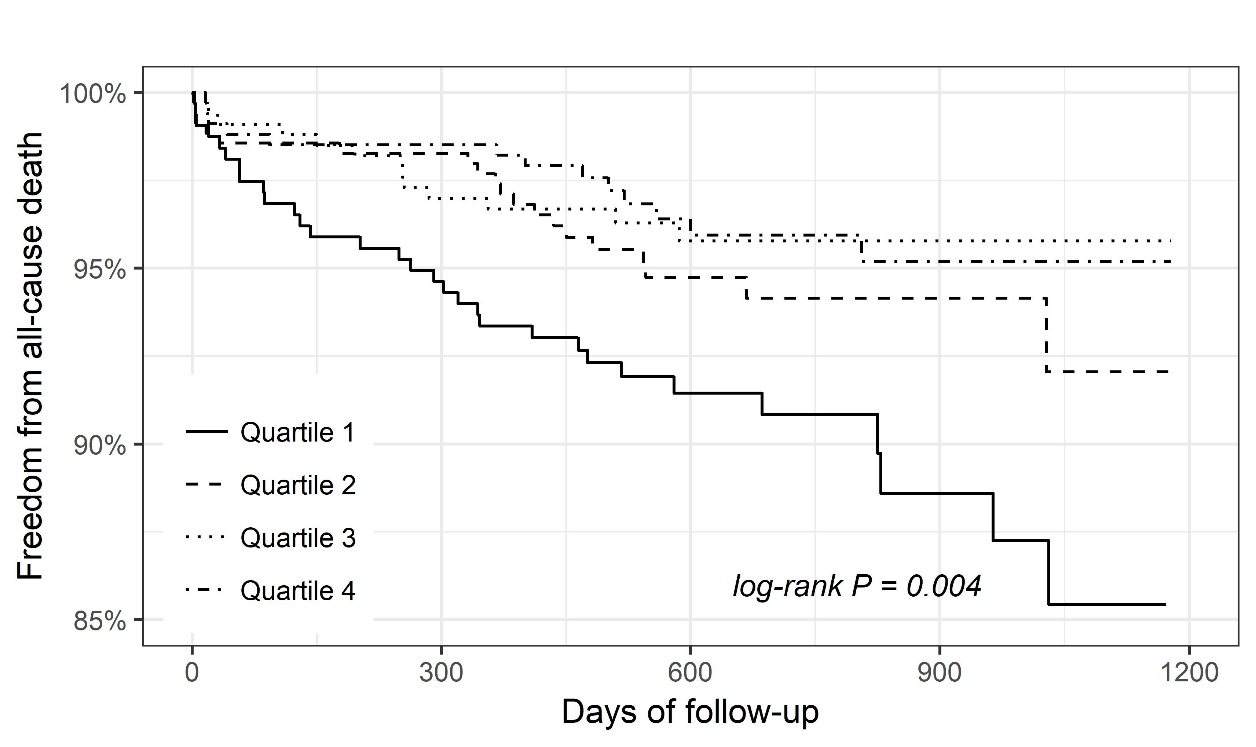


Supplemental Figure S3 (a): Kaplan-Meier curves comparing survival among serum calcium quantiles in acute coronary syndrome (ACS) patients with a history of hypertension. The curves of the quartiles of calcium differed significantly (log-rank *P*=0.004), and the patients in the lowest calcium quartile had a highly significant elevated cumulative incidence of mortality.


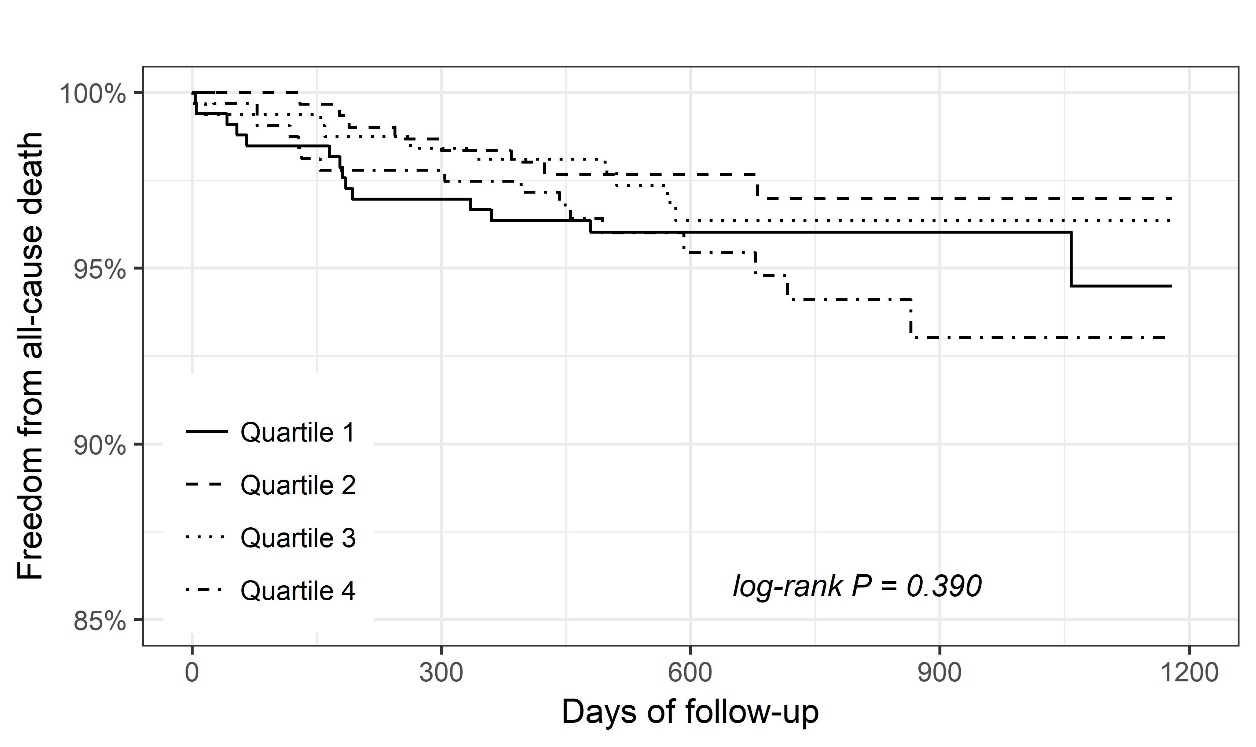


Supplemental Figure S3 (b): Kaplan-Meier curves comparing survival among serum calcium quantiles in acute coronary syndrome (ACS) patients with no history of hypertension. The curves of the quartiles of calcium were not significantly different (log-rank *P*=0.390).

Supplemental Table S1. Relationships between risk factors and mid-term mortality in patients with acute coronary syndrome by univariate analysis.

| Factor | *β* | *SE* | *z value* | *P* | HR(95% CI) |
| --- | --- | --- | --- | --- | --- |
| **Demographics** |  |  |  |  |  |
| Age | 0.07 | 0.01 | 7.69 | <0.001 | 1.07 (1.05, 1.09) |
| Men | 0.28 | 0.19 | 1.51 | 0.132 | 1.32 (0.92, 1.91) |
| Current smoking | -0.26 | 0.18 | -1.41 | 0.159 | 0.78 (0.54, 1.11) |
| Current alcohol use | -0.38 | 0.25 | -1.51 | 0.132 | 0.69 (0.42, 1.12) |
| **Medical history** |  |  |  |  |  |
| Hypertension | 0.44 | 0.18 | 2.36 | 0.018 | 1.55 (1.08, 2.22) |
| Diabetes mellitus | 0.18 | 0.21 | 0.85 | 0.396 | 1.19 (0.79, 1.79) |
| Stroke | 0.76 | 0.20 | 3.74 | <0.001 | 2.14 (1.44, 3.19) |
| CHD | 0.49 | 0.22 | 2.26 | 0.024 | 1.63 (1.07, 2.48) |
| Previous PCI | 0.35 | 0.30 | 1.17 | 0.244 | 1.43 (0.79, 2.58) |
| **In-hospital complications** |  |  |  |  |  |
| Acute heart failure | 1.10 | 0.19 | 5.91 | <0.001 | 3.00 (2.08, 4.32) |
| Acute arrhythmia | 1.11 | 0.35 | 3.20 | 0.001 | 3.03 (1.54, 5.98) |
| AV block | 0.72 | 0.28 | 2.55 | 0.011 | 2.06 (1.18, 3.59) |
| **Medication on admission** |  |  |  |  |  |
| ACEI/ARB | -0.16 | 0.19 | -0.81 | 0.418 | 0.86 (0.59, 1.25) |
| Beta-blocker | -0.30 | 0.18 | -1.66 | 0.097 | 0.74 (0.52, 1.06) |
| Aspirin | -1.04 | 0.28 | -3.78 | <0.001 | 0.35 (0.21, 0.61) |
| Statin | -0.58 | 0.31 | -1.89 | 0.059 | 0.56 (0.31, 1.02) |
| **Main diagnosis** |  |  |  |  |  |
| STEMI | —— | —— | —— | —— | —— |
| NSTEMI/UA | -0.02 | 0.18 | -0.09 | 0.932 | 0.99 (0.69, 1.41) |
| **Reperfusion strategy** |  |  |  |  |  |
| Coronary angiography | -0.99 | 0.19 | -5.21 | <0.001 | 0.37 (0.26, 0.54) |
| PCI | -0.86 | 0.19 | -4.52 | <0.001 | 0.43 (0.29, 0.62) |
| Thrombolysis | -0.17 | 0.46 | -0.38 | 0.705 | 0.84 (0.34, 2.06) |
| **Laboratory results** |  |  |  |  |  |
| Hemoglobin | -0.41 | 0.07 | -5.62 | <0.001 | 0.66 (0.57, 0.76) |
| Albumin | 0.08 | 0.06 | 1.35 | 0.179 | 1.08 (0.97, 1.21) |
| BUN | 0.20 | 0.04 | 5.34 | <0.001 | 1.22 (1.13, 1.31) |
| Fasting glucose | 0.04 | 0.05 | 0.72 | 0.475 | 1.04 (0.94, 1.15) |
| eGFR | -0.67 | 0.08 | -8.49 | <0.001 | 0.51 (0.44, 0.60) |
| Uric acid | 0.20 | 0.09 | 2.40 | 0.017 | 1.23 (1.04, 1.45) |
| Serum calcium | -0.47 | 0.08 | -5.64 | <0.001 | 0.63 (0.53, 0.74) |
| Serum phosphate | 0.10 | 0.04 | 2.65 | 0.008 | 1.10 (1.03, 1.18) |
| Serum magnesium | 0.11 | 0.05 | 2.35 | 0.019 | 1.12 (1.02, 1.22) |
| Serum potassium | 0.13 | 0.06 | 2.35 | 0.019 | 1.14 (1.02, 1.27) |
| Serum sodium | -0.08 | 0.06 | -1.22 | 0.222 | 0.92 (0.82, 1.05) |
| Serum chloride | -0.09 | 0.07 | -1.26 | 0.206 | 0.92 (1.05, 1.09) |
| **Echocardiography results** |  |  |  |  |  |
| LVEF | -0.56 | 0.09 | -6.14 | <0.001 | 0.57 (0.48, 0.69) |
| LAD | 0.35 | 0.09 | 3.99 | <0.001 | 1.42 (1.20, 1.69) |
| LVEDD | 0.27 | 0.08 | 3.26 | 0.001 | 1.31 (1.11, 1.54) |
| IVST | 0.09 | 0.04 | 2.38 | 0.017 | 1.09 (1.02, 1.18) |
| LVPW | -0.20 | 0.20 | -0.98 | 0.327 | 0.82 (0.56, 1.22) |

Abbreviations: ACEI, angiotensin-converting enzyme inhibitors; ARB, angiotensin receptor blockers; BUN, blood urea nitrogen; CHD, Coronary heart disease; eGFR, estimated glomerular filtration rate; IVST, interventricular septum thickness; LAD, left atrial diameter; LVEDD, left ventricular end diastolic diameter; LVEF, left ventricular ejection fraction; LVPW, left ventricular posterior wall thickness; NSTEMI, non-ST-segment elevation myocardial infarction; PCI, percutaneous coronary intervention; and STEMI, ST-segment elevation myocardial infarction.

Supplemental Table S2: Discriminatory ability of serum calcium for mid-term mortality according to the C-Index and NRI.

| Model | C-index | *P value* | NRI | *P value* |
| --- | --- | --- | --- | --- |
| Established risk factors | 0.75 | Ref | Ref | Ref |
| Established risk factors + serum calcium | 0.77 | 0.042 | 0.22 | 0.025 |

Established risk factors included age (continuous), sex, history of hypertension, history of stroke, history of coronary heart disease, complicated heart failure, complicated arrhythmia, complicated atrioventricular block, aspirin on admission, percutaneous coronary intervention, coronary angiography, hemoglobin (continuous), uric acid (continuous), eGFR (continuous), serum phosphate (continuous), serum magnesium (continuous), serum potassium
